# 1836. Disparities in COVID-19 Vaccination Coverage Among a Large Patient Population in a Network of Community-Based Healthcare Organizations

**DOI:** 10.1093/ofid/ofad500.1665

**Published:** 2023-11-27

**Authors:** Holly C Groom, Frances Biel, Bradley Crane, Evelyn Sun, Joanna Georgescu, Eric Weintraub, Michael McNeil, Amelia Jazwa, Constance Owens, Allison L Naleway, Teresa Schmidt

**Affiliations:** Kaiser Permanente Center for Health Research, Portland, Oregon; OCHIN, Portland, Oregon; Kaiser Permanente Center for Health Research, Portland, Oregon; OCHIN, Portland, Oregon; OCHIN, Portland, Oregon; Centers for Disease Control and Prevention, Atlanta, GA; CDC, Atlanta, Georgia; CDC, Atlanta, Georgia; OCHIN, Portland, Oregon; Kaiser Permanente Center for Health Research, Portland, Oregon; OCHIN, Portland, Oregon

## Abstract

**Background:**

There are known disparities in U.S. COVID-19 vaccination by race, ethnicity, and rurality, but there is limited information on national trends in vaccine uptake in a large, racially diverse population spanning all ages. Here we describe COVID-19 vaccination coverage in a large US population accessing care in a national network of community-based healthcare organizations (OCHIN).

**Methods:**

Within the OCHIN network, we identified all individuals aged 6 months and older who had at least one completed encounter in an OCHIN facility across 26 U.S. states since becoming age-eligible for the COVID-19 vaccine, through 2022. Patients’ COVID-19 vaccination status was assessed from the OCHIN Electronic Health Record which includes data from state immunization information systems. Individuals were categorized as vaccinated with the primary series if ≥2 appropriately spaced doses of either mRNA monovalent product, or a single dose of Johnson & Johnson® vaccine, were documented. Coverage was assessed by age groups based on the roll-out of COVID-19 vaccine recommendations (6 months-4 years; 5-11 years, 12-15 years, 16+ years).

**Results:**

The cohort of over 3.3 million identified as Hispanics (37%), non-Hispanic (NH) Whites (31%), NH Blacks (15%), and NH Asians (7%); 44% of whom were Medicaid-enrolled, 19% without health insurance, and 53% with a household income below 100% of the federal poverty level.

The proportion fully vaccinated increased incrementally with age and ranges from a low of 7.2% (6 months through 4 years) to a high of 63.4% (75 years and older); consistently lower than national coverage estimates across age groups (**Figure 1**). Patterns are consistent across age groups, where coverage is highest among NH Asians (range: 13%-64%) and lowest for NH Blacks (3%-40%) **(Figure 2)**; and lowest among the people without insurance (7%-41%). Those with ≥ 1 prior influenza vaccine since 2019 had higher coverage than those without, most notably among those aged 16+ years (71% vs. 16% fully vaccinated, respectively). (**Figure 3**)

**Figure 1.**
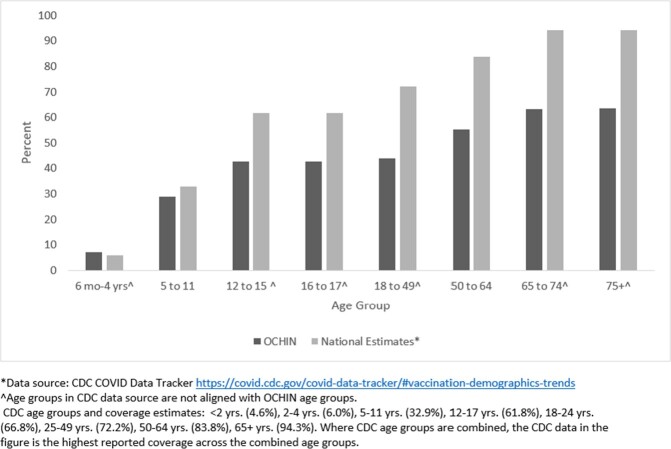
COVID-19 Vaccine Primary Series Coverage, OCHIN vs. CDC National Estimates*, OCHIN, December 2022

**Figure:**
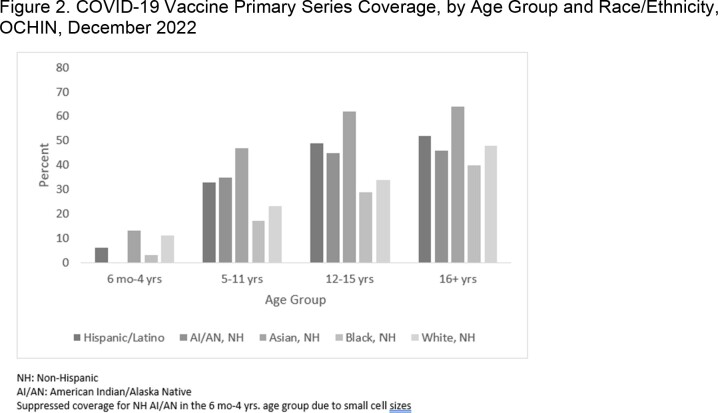

**Figure:**
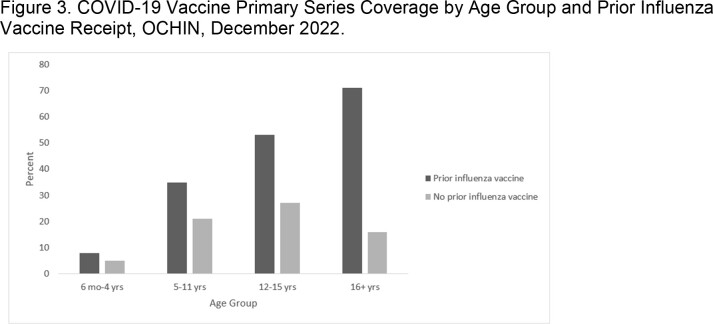

**Conclusion:**

We identified disparities in primary series COVID-19 vaccine coverage by age, race and ethnicity, household income, insurance status, and by prior influenza vaccination within this large, diverse population accessing care in community-based healthcare organizations.

**Disclosures:**

**All Authors**: No reported disclosures

